# Advances in Water, Air and Soil Pollution Monitoring, Modeling and Restoration

**DOI:** 10.3390/toxics12040244

**Published:** 2024-03-26

**Authors:** Alina Bărbulescu, Lucica Barbeş, Cristian Ștefan Dumitriu

**Affiliations:** 1 Department of Civil Engineering, Transilvania University of Brașov, 5 Turnului Str., 500152 Brașov, Romania; alina.barbulescu@unitbv.ro; 2 Department of Chemistry and Chemical Engineering, “Ovidius” University of Constanța, 124 Mamaia Bd., 900527 Constanța, Romania; 3 Doctoral School of Biotechnical Systems Engineering, Politehnica University of Bucharest, 313, Splaiul Independenţei, 060042 Bucharest, Romania; 4 Faculty of Mechanical Engineering and Robotics in Constructions, Technical University of Civil Engineering, Calea Plevnei 59, 021242 Bucharest, Romania

Global pollution demands continuous attention and concerted efforts to reduce its effects. Every day, our planet faces increasing pressure from various sources, including industrial activities, urbanization, agriculture, and waste generation. In this context, the articles featured in this Special Issue shed light on the multifaceted nature of environmental pollution and provide innovative approaches for its monitoring and modeling, proposing solutions for restoration. Several articles delve into the complexity of pollution assessment, providing insights into the impact of pollutants on environmental health and human wellbeing. Studies focusing on degradation processes emphasize the importance of understanding pollution’s ecological consequences. Therefore, a key theme of these investigations is the urgent need for effective mitigation measures to address environmental restoration. Moreover, the articles provide valuable guidance for policy makers, practitioners, and researchers.

As academic editors, we are particularly excited to see this collection’s diverse topics. We hope that the presented discoveries will inspire further interdisciplinary collaboration and innovative solutions to the challenges posed by environmental pollution. Eleven papers were selected for inclusion in this issue after the peer review process of the twenty-three submitted manuscripts. The complexity of this Special Issue lies in interpreting the multifaceted interactions between various environmental parameters and developing effective pollution monitoring and management strategies.

In the article “Polycyclic Aromatic Hydrocarbons (PAHs) in the Dissolved Phase, Particulate Matter, and Sediment of the Sele River, Southern Italy: A Focus on Distribution, Risk Assessment, and Sources”, Montuori et al. present the findings on the concentrations and composition of PAHs in the Sele River, Italy. Low-molecular-weight (LMW) PAH levels were notably elevated in water samples, while high-molecular-weight (HMW) PAHs were predominant in sediment samples. Analysis of the PAHs’ diagnostic ratio indicates that the primary sources were pyrolytic, suggesting a significant contribution from vehicle emissions and combustion processes. The concentrations of numerous individual PAHs at various sites surpassed environmental risk limits (ERLs) and threshold effect levels (TELs) [1], occasionally resulting in adverse environmental impacts. However, the toxic equivalent concentration (TEQ) of carcinogenic PAHs shows a definite carcinogenic risk in the Sele River basin. Hence, continuous monitoring of Sele River waters is imperative as PAH contamination could affect aquatic ecosystems.

The article “Occurrence and Distribution of Persistent Organic Pollutants (POPs) from Sele River, Southern Italy: Analysis of Polychlorinated Biphenyls and Organochlorine Pesticides in a Water–Sediment System” investigates the pollution characteristics, spatiotemporal variation, source, and potential ecological risk of PCBs and OCPs in the Sele River, including their contribution to the Tyrrhenian Sea. Sediment samples exhibited higher levels of these contaminants compared to those in the water bodies, DP, and SPM, indicating that suspension processes and sedimentation are the primary mechanisms at work in the Sele River. The data showed that industrial processes were the primary source of PCBs. Risk assessment revealed elevated PCB risk factors at the mouth of the Sele River and 500 m south, while levels were lower at other sites. In contrast, OCP ratios were generally lower, with most analytes showing a risk quotient (RQ) below 1. Consequently, regular monitoring of pollution in the Sele River and its estuary is necessary to evaluate ecological risks over time. These findings enhance our understanding of Sele River water quality and inform environmental monitoring, applications of sediment quality guidelines, and ecological risk assessments [2]. It is expected that establishing a comprehensive database for various pollution factors and including more emerging contaminants in river ecosystem risk assessments will be crucial. Moreover, this study’s results will aid in preventing future contamination of the Sele River’s water system by PCBs and OCPs, thereby strengthening prevention and pollution control measures against future risks. The results will help policy makers identify high-risk pollutant areas, improve environmental protection regulations, and raise public awareness of their importance. 

The research study “Health Risk Assessment of PAHs from Estuarine Sediments in the South of Italy” introduces, for the first time, an evaluation of the carcinogenic risk posed to human health by dermal and ingestion exposure to polycyclic aromatic hydrocarbons (PAHs) present in sediments within the primary surface water streams of the Campania Region, located in southern Italy. It offers insights into the concentrations, spatial distribution, and composition profiles of PAHs found in sediments collected near the estuaries of the Sele, Sarno, and Volturno Rivers. The findings suggest that the risk of cancer resulting from oral exposure to PAHs in estuarine sediments [3]—quantified as incremental lifetime cancer risk (ILCR ingestion)—is low, unlike the risk associated with accidental skin exposure, which is moderate. The results underscore the need to continuously evaluate the carcinogenic risk to human health arising from dermal and oral exposure to PAHs and ongoing monitoring of PAH concentrations in surface water sediments within the Campania Region. Therefore, this study is a foundation for future investigations to comprehensively assess the carcinogenic risk to human health due to PAH exposure to inform pollution prevention measures, ecological restoration strategies for rivers, and the preservation of our overall wellbeing.

In their work titled “Modeling the Chlorine Series from the Treatment Plant of Drinking Water in Constanta, Romania”, Bărbulescu and Barbeş introduced four alternative approaches for modeling monthly free chlorine residual concentration series from PCTP using decomposition, Holt–Winters, and SARIMA models. A key novelty lies in employing econometric models in engineering, thereby expanding upon previous studies on the water quality, which had primarily used statistical modeling [4,5]. Research in Romania has been limited in this field, with it being primarily experimental or presenting basic statistics without correlations. This article fills this gap in research, which is particularly crucial given the importance of monitoring chlorine concentration to avoid exceeding regulatory limits and potential public discontent due to changes in drinking water taste and smell. However, these models are recommended for short-term predictions without continuous updating. Automating chlorine concentration monitoring can improve dosage and forecasting accuracy. Additionally, future studies should consider incorporating risk factors and addressing water quality deterioration to ensure constant monitoring and intervention in the water treatment process.

The article “Assessing the Efficiency of a Drinking Water Treatment Plant Using Statistical Methods and Quality Indices” by Bărbulescu and Barbeș introduces various indicators utilized in a case study to assess the effectiveness of a water treatment plant. While individual indicators highlight efficiency concerning specific water parameters and underscore issues that may arise during particular periods or regarding specific parameters, cumulative indicators evaluate overall efficiency over time, considering all parameters. This study revealed that individual efficiencies are sensitive to fluctuations in effluent values compared to influent values, even if they fall within maximum allowable variation (MAV) limits. Consequently, cumulative indices can be significantly influenced when very low values contribute to their calculation. Weighted cumulative indices consistently differ from the average ones. However, given the significance of each water parameter and the imperative of maintaining high water quality standards, they must be considered.

The study paves the way for aligning evaluations of environmental pollution with sustainability objectives based on objective criteria [6,7,8]. Future research will explore opportunities to enhance the presented indices and establish a system that promptly implements necessary corrective measures upon issue detection. Additionally, a procedural framework must be devised to address the outliers’ existence because these values introduce considerable biases in the indices computation.

The paper “Determinants Analysis Regarding Household Chemical Indoor Pollution” highlights the need for more comprehensive research on indoor household pollution among the general population. Despite being aware of the harmful effects of certain habits, it remains difficult for people to adopt behaviors that help reduce indoor pollution. Therefore, there is an urgent need for training programs that can target individuals with poor indoor habits, such as singles, smokers, and those with lower education, to help them improve their practices and minimize exposure to indoor pollutants [9]. Additionally, educational initiatives are needed to reinforce the importance of good practices among individuals who already exhibit positive attitudes and behaviors, such as those in committed relationships and non-smokers. Although there are behavior and attitude correction programs for highly educated youth, there is still a gap in translating this knowledge into practical measures to effectively address indoor chemical pollution.

The article “A New Method for Ecological Risk Assessment of Combined Contaminated Soil” indicates that the ecological risk assessment of combined polluted soil has traditionally relied on the risk screening value (RSV) of individual pollutants, but this approach has notable limitations. It overlooks the influence of soil properties and fails to consider interactions among different pollutants. This study evaluated the ecological risks associated with 22 soils from 4 smelting sites using toxicity tests involving soil invertebrates. In addition to the RSV-based assessment, a novel method was developed.

This new approach introduced a toxicity effect index (EI) to standardize the toxicity effects of various endpoints, enabling comparisons across different toxicity measures. Furthermore, an ecological risk probability assessment method (RP) was devised based on the cumulative probability distribution of EI. A significant correlation was observed between the RP based on EI and the Nemerow ecological risk index (NRI) [10] based on RSVs (*p* < 0.05). Additionally, the new method facilitates the visual representation of probability distributions for various toxicity endpoints, aiding risk managers in devising more effective risk management strategies to safeguard critical species. This innovative method is poised to integrate with a sophisticated dose–effect relationship prediction model constructed using machine learning algorithms, offering a fresh approach to the ecological risk assessment of combined contaminated soil.

The article “Evaluating the Contamination by Indoor Dust in Dubai” presents an analysis of metal enrichment levels in indoor dust collected from various locations in Dubai, utilizing multivariate statistics and pollution indices. The research addresses a significant gap in understanding indoor pollution caused by dust in a region prone to frequent dust storms. Results indicated that the highest enrichment factors (for Ca, Cu, Mg, and Fe) were attributed to soil lithology and industrial activities, particularly mining, with dust transportation over long distances during dust storms. Two novel pollution indices, CPI and AWI, were introduced and applied to assess contamination levels at observation sites. Classification of sites based on PLI, CPI, AWI, and the Nemerow index [11] differed from classifications based on raw data series, with two sites falling into distinct clusters in each classification. This study also suggests a promising research direction using different classification data sets. Notably, eliminating elements with concentrations significantly below warning limits from the data set resulted in more realistic classifications. The future of this research aims to develop a methodology for cross-validating clustering findings using supplementary selection criteria and decision trees. Employing various clustering algorithms on raw data series, pollution index series, and stability criteria will be crucial for identifying consistently similar series within the data set. Overall, this study provides valuable insights into indoor dust pollution in Dubai and lays the groundwork for further research, with potential implications for pollution management and public health.

The paper “Toxicity Risk Assessment Due to Particulate Matter Pollution from Regional Health Data: Case Study from Central Romania” presents the health implications of elevated levels of PM10 and PM2.5 above the average limits recommended by Romanian legislation and the World Health Organization (WHO) in the Central Region of Romania. The findings underscore the significant risk of prolonged exposure to airborne fine particulate matter, commonly found in urban areas, on cardiovascular health. According to the health impact assessment conducted in this study, adhering to the new WHO limits could yield substantial benefits in reducing mortality rates in the Central Region of Romania. Specifically, adopting these limits could reduce approximately 196 deaths on average and an increase in life expectancy by approximately 5.3 months due to lower PM2.5 levels. Furthermore, there could be a decrease of roughly 190 deaths on average, corresponding to a 3.5-month increase in life expectancy related to cardiovascular mortality.

These results highlight the urgent need to mitigate the health risks associated with pollutants’ exposure [12,13] by implementing the new WHO-recommended limits in Romanian regulations. However, it is essential to acknowledge the limitations of this study, such as data gaps, especially regarding PM2.5, which may affect the accuracy of the PM2.5/PM10 ratio estimation. As a future direction, expanding the scope of this study to include other pollutants is crucial.

The paper “Prediction for Cyanobacterial Blooms Using Environmental Variable Selection and Data Resampling” introduce a series of processes to enhance the prediction accuracy of algal alert levels in the BJR by using observed data, feature selection techniques, and resampling methods to construct two machine learning models [14,15]. The primary objective of this study was to develop a prediction model for algal alert levels in reservoirs using readily available data from national monitoring stations. The proposed model, which incorporates feature selection and resampling methods, is anticipated to benefit engineers and decision makers in managing algal blooms in watershed areas, including inland weirs. This model will facilitate the development of effective strategies and regulations for constructing and operating these reservoirs.

The article titled “Application of Machine Learning in Modeling the Relationship between Catchment Attributes and Instream Water Quality in Data-Scarce Regions”, highlights the efficacy of machine learning methods [16] in predicting and evaluating water quality parameters within a catchment area. Among these methods, the random forest (RF) model is the most effective, providing a robust tool for accurate and efficient water quality assessment. While certain models may exhibit shortcomings in specific criteria, a nuanced assessment using relative criteria such as accuracy (R^2^) and mean absolute percentage error (MAPE) underscores the overall robustness of predictive models. Evaluation of R^2^ values indicates satisfactory performance across all models except pH prediction. Despite slightly elevated MAPE values in five models (SAR, Na^+^, SO_4_, Cl^−^, TDS), the primary research objective—understanding the significance of individual input variables within data constraints—was achieved. This accomplishment lays the groundwork for selecting and implementing optimal models from a broader spectrum of machine-learning techniques.

Integrating these research findings into decision-making processes offers transformative opportunities for strategic resource allocation and environmental impact mitigation. Furthermore, this integration empowers decision makers to adopt targeted strategies for promoting environmental sustainability, contributing to the broader objective of nurturing resilient water ecosystems. This approach signifies a practical pathway towards achieving a delicate balance between human activities and ecological preservation, actively promoting sustainable water ecosystems.

Finally, we sincerely thank the authors, reviewers, and editorial team for their invaluable contributions to this Special Issue. The research presented here will catalyze continued progress in environmental science and contribute to our ongoing efforts to safeguard our precious natural resources. Working together, we can strive towards a cleaner, healthier, and more sustainable planet for future generations.
